# Adopting machine learning to predict breast cancer patients adherence with lifestyle recommendations and quality of life outcomes

**DOI:** 10.3389/fdgth.2025.1645233

**Published:** 2025-11-06

**Authors:** Anna Crispo, Maria Elisabetta Pagnano, Agnese Bonfigli, Leandro Pecchia, Assunta Luongo, Giuseppe Porciello, Sergio Coluccia, Melania Prete, Luca Bacco, Sara Vitale, Elvira Palumbo, Paolo Giaccone, Rosa Pica, Maria Grimaldi, Marco Cascella, Ernesta Cavalcanti, Anita Minopoli, Michelino De Laurentiis, Massimo Libra, Jerry Polesel, Samuele Massarut, Egidio Celentano, Livia S. A. Augustin

**Affiliations:** 1 Epidemiology and Biostatistics Unit, Istituto Nazionale Tumori - IRCCS, “Fondazione G. Pascale”, Naples, Italy; 2 Research Unit of Intelligent Health-Technologies, Department of Engineering, Università Campus Bio-Medico di Roma, Rome, Italy; 3 Fondazione Policlinico Universitario Campus Bio-Medico, Rome, Italy; 4 Branch of Medical Statistics, Biometry and Epidemiology “G. A. Maccacaro”, Department of Clinical Sciences and Community Health, Università degli Studi di Milano, Milan, Italy; 5 Research Unit of Computer System and Bioinformatics, Department of Engineering, Università Campus Bio-Medico di Roma, Rome, Italy; 6 Anesthesia and Pain Medicine, Department of Medicine, Surgery and Dentistry “Scuola Medica Salernitana”, University of Salerno, Baronissi, Italy; 7 Division of Laboratory Medicine, Istituto Nazionale Tumori - IRCCS, “Fondazione G. Pascale”, Naples, Italy; 8 Division of Breast Medical Oncology, Department of Breast and Thoracic Oncology Director, Istituto Nazionale Tumori - IRCCS, “Fondazione G. Pascale”, Naples, Italy; 9 Department of Biomedical and Biotechnological Sciences, University of Catania, Catania, Italy; 10 Cancer Epidemiology Unit, Centro di Riferimento Oncologico di Aviano (CRO), IRCCS, Aviano, Italy; 11 Breast Surgical Oncology Unit, Centro di Riferimento Oncologico di Aviano (CRO), IRCCS, Aviano, Italy

**Keywords:** breast cancer, machine learning, missing data, diet, health-related quality of life

## Abstract

**Introduction:**

Healthy lifestyle behaviors and improved quality of life have been associated with better prognoses in breast cancer survivors. However, sustaining behavioral changes remains challenging; therefore, identifying effective components of lifestyle education programs is essential to enhance adherence, improve quality of life, and facilitate their integration into clinical practice. This study aimed to predict patient adherence to a lifestyle intervention of diet, physical activity, and vitamin D supplementation and to forecast the most frequent Health-Related Quality of Life over the subsequent three measurements.

**Methods:**

A total of 316 breast cancer survivors were included in the analysis. Adherence was modeled as a multi-label time series classification task, with compliance recorded on a three-point scale for each treatment component at quarterly intervals over one year. Health-Related Quality of Life was predicted by evaluating first-year adherence data to estimate the mean score over the subsequent three measurements.

**Results:**

The dataset was split into 70% for training and 30% for evaluation. Random forest classifiers were employed for adherence prediction, achieving accuracy of up to 81%. An XGBoost regressor was used for Health-Related quality of life prediction, and it was compared to a baseline linear regression model. XGBoost demonstrated superior predictive performance, achieving an R-squared value of 0.62.

**Discussion:**

Our findings highlight the promise of machine learning techniques in supporting personalized medicine. Advanced predictive models may aid in identifying patients at risk of non-adherence, enabling early interventions, and improving long-term outcomes through tailored lifestyle strategies for breast cancer survivors.

## 1 Introduction

According to the latest data from Global Cancer Observatory (GLOBOCAN 2022) breast cancer (BC) is the second most common cancer worldwide and the fourth leading cause of cancer-related death.

In 2022, there were 2.3 million new cases and 666,000 deaths (1), and the number of BC survivors continues to increase, with 5-years survival rates at around 80% (2). Therefore, there is a growing interest in clarifying how cancer, treatment, and lifestyle factors affect BC survivors (3). In this regard, evidence indicates that modifiable risk factors such as weight gain and physical inactivity, both prior to and following diagnosis and treatment could negatively affect BC prognosis (4, 5). Furthermore, strong evidence suggests that intervention studies aimed at increasing physical activity may improve the quality of life in BC patients (6, 7). In this context, the assessment of the adherence to a lifestyle modification program plays a central role. Dietary intervention adherence is evaluated using validated tools (food records, food frequency questionnaires, blood tests), and goal attainment scales. Physical activity adherence is evaluated through validated tools that include digital technology (i.e., steps count), and goal attainment scales. These latter can be used to quickly obtain information on adherence to a lifestyle program over time and to understand the impact on patient-reported outcomes (PRO), specifically Health-Related Quality of Life (HRQoL) in conjunction with validates tools (4, 8). According to the World Health Organization (WHO), HRQoL is an important self-perceived parameter of patients' general health, providing information on physical, psychological and emotional characteristics, and social appearance (9). HRQoL assessment in cancer patients provides important information to clinicians, representing a crucial endpoint in health and clinical research. In this regard, evidence indicates that BC survivors may experience several physical and mental disorders including pain, fatigue and anxiety (10–15). Moreover, the long-term effects of cancer and its treatment could negatively influence cognitive function, including symptoms such as anxiety, depression, fear of recurrence, psycho-physical stress, lack of concentration, memory loss, disease-related cognitive fog (“chemobrain”) and sleep disturbances (16–18). A growing number of studies evaluated the role of healthy dietary patterns on HRQoL in BC survivors. Evidence from prospective cohort studies showed that higher consumption of a vegetables and fruits-based dietary pattern is associated with better scores in global health status/quality of life, physical functioning, emotional functioning and cognitive functioning, as well as fewer symptoms of nausea and vomiting, dyspnea, insomnia, loss of appetite, constipation and diarrhea (19).

In Italy, two cross-sectional investigations from DEDiCa study indicate that higher adherence to the Mediterranean diet in a subgroup of BC survivors is associated with better aspects of quality of life, specifically higher physical functioning, better sleep, lower pain, and generally higher well-being (20) as well as higher overall quality of life (21). As the impact of diet and lifestyle on HRQoL in BC survivors becomes increasingly evident, integrating innovative tools such as Machine learning (ML) may further enhance our ability to monitor, predict, and personalize these interventions.

Within this context, artificial intelligence is increasingly gaining importance in patient-reported clinical outcomes evaluation and adherence to lifestyle interventions, as well as in other areas of research (22). ML approaches, and related predictive analytics are now used to enhance cancer diagnosis, forecast treatment outcomes, and inform therapy plans (23). One of the key objectives of oncological research is the identification of reliable and validated methodologies for predicting risk, enabling early diagnosis, assessing clinical prognosis, and understanding disease-related behaviors in cancer patients (24).

This study aims to apply advanced ML techniques, to model and predict health-related behaviors in women diagnosed with BC who are enrolled in the DEDiCa study (25). The patients attended study visits every three months from the baseline (BL) visit and were evaluated on their adherence to the treatment. We aimed to address two primary research questions (RQ): the RQ1 was to predict the most frequent pattern of patient compliance with recommendations regarding diet, exercise, and vitamin D supplementation across the follow-up points (M3, M6, M9, M12) over the subsequent 9 months. This prediction was based on compliance data collected from third (M3) up to 12th month of follow-up (M12) and on patients' BL clinical and demographic characteristics. RQ2 was to forecast quality of life outcomes by using HRQoL measures assessed from BL to M12 in addition with BL patients' characteristics.

The target variable was the average HRQoL across the subsequent 9 months of follow-up. By analyzing the trajectories of HRQoL measures from the study's early phase, these approaches seek to gain deeper insights into how these behaviors evolve, helping to tailor interventions that support sustained lifestyle changes and ultimately improve patient outcomes.

## 2 Materials and methods

### 2.1 The trial

DEDiCa study is an Italian multicenter randomized controlled trial, started in 2016 and approved by the Ministry of Health, Italian Drugs Agency-AIFA (EudraCT 2015-005147-14), and the Ethics Committees of the participating centers (ClinicalTrials.gov identifier https://clinicaltrials.gov/ct2/show/NCT02786875). The primary endpoint of DEDiCa study is to evaluate the effect of an intervention combining diet, physical activity (PA), and vitamin D supplementation on BC recurrence and disease-free survival. While the secondary endpoint includes improvements in cardio metabolic health and HRQoL (25). The patients observed in this study were recruited in cancer units of research hospitals in Italy: Istituto Nazionale Tumori IRCCS Fondazione “G. Pascale” (Naples), Azienda Ospedaliera per l'emergenza Cannizzaro (Catania), Ospedale San Vincenzo di Taormina (Taormina), Centro Riferimento Oncologico—CRO (Aviano). Eligible participants were women aged ≥30 <75 years with a primary diagnosis of histologically confirmed BC (stages I-III, without metastasis), within 12 months from diagnosis (25), who can understand and sign informed consent, as well as adhere to the study protocol. Patients with other malignancies, severe renal failure, hypercalcemia, kidney stones, granulomatous diseases, or sarcoidosis are excluded.

### 2.2 Data collection

Data on anthropometric measurements, dietary intake, PA, HRQoL, and blood parameters [including serum 25(OH)D] were collected at BL and during follow-up visits (M3, M6, M9, M12).

At the BL visit, anamnesis, clinical data, and information on vitamin D supplementation were also recorded. At each follow-up, trained nutritionists collected data on ongoing pharmacological treatments, clinical notes, and adherence to lifestyle modifications. Dietary intake was assessed using a 7-day food diary and were processed using a professional software WinFood© (version 3.9.0; Medimatica Srl Italy), which utilized two Italian nutrition databases, CREA (Council for Agricultural Research and Economics), and BDA (Food Composition Database for Epidemiological Studies in Italy). While PA was monitored via an electronic pedometer (Omron Walking Style IV, HJ-325-EB—OMRON Healthcare Customer Service Europe© 2025) and a structured questionnaire. Serum 25(OH)D concentrations were measured using the chemiluminescent immunoassay (CLIA) method with DiaSorin kits on the Liaison XL analyzer (DiaSorin S.p.A., Italy). Samples, collected in anticoagulant-free Vacutainer tubes (Becton, Dickinson and Co., Franklin Lakes, NJ, USA), were analyzed within 2 h of blood collection or thawing. All blood samples were processed in the reference laboratory (Istituto Nazionale Tumori IRCCS Fondazione “G. Pascale” Naples) under standard quality control procedures (25). Vitamin D dosage was monitored and adjusted at follow-ups based on 25(OH)D levels to meet group targets.

### 2.3 Adherence to dietary intervention in Bc patients

Daily foods intake and portion sizes were assessed using food diaries. If necessary, nutritionists supplemented the information with targeted questions. Foods were classified as recommended or discouraged according to the principles of the Mediterranean diet, with specific adaptations for breast cancer survivors, in line with the World Cancer Research Fund (WCRF) recommendations (https://www.wcrf.org/cancer-prevention-recommendations/). Diet adherence (AD_DIET) was classified on a three-point scale: 1 point for poor compliance (i.e., adherence to <50% of dietary advice), 2 points for moderate compliance (i.e., adherence from 50% to <80% of dietary advice), and 3 points for higher adherence (ranging from 80% to 100%).

### 2.4 Adherence to physical intervention in Bc patients

Adherence to PA recommendations (AD_PA) was assessed quarterly by calculating the average number of steps taken by participants during the week prior to the visit. Patients were encouraged to walk briskly for half an hour per day. PA adherence was classified on a scale from 1 to 3: if patients increased the average number of steps by 4,000−5,000 compared to BL, a score of 3 was assigned; if the average number of steps was half of the BL, a score of 2 was assigned. Otherwise, a score of 1 was assigned.

### 2.5 Adherence to vitamin D intake recommendations

Vitamin D dosage was monitored every 3 months via serum 25(OH)D and adjusted to achieve sufficiency (between 30 ng/mL and 60 ng/mL). Adherence to vitamin D supplementation (AD_VITD) was evaluated on a 3-point scale: 3 points were assigned if the patient achieved sufficient vitamin D levels when initially deficient; 2 points were given if the patient frequently forgot to take the supplement, resulting in inconsistent intake. While 1 point was assigned if the patient did not report improvement in vitamin D status.

### 2.6 Quality of life assessment

HRQoL was assessed through a validate questionnaire, the European Quality of Life 5 Dimensions 3 Level (EQ-5D-3l) (26). It gives a non-cancer-specific measure of generic health status that includes a descriptive system comprising five dimensions (mobility, self-care, usual activities, pain or discomfort, and anxiety or depression) and three levels of perceived problems (1 for no problems, 2 for some problems, and 3 for extreme problems). A unique health index score is calculated by applying an algorithm that sues coefficients (called weights) to each value of the levels for each dimension; the Italian Model was used to estimate EQ-5D-3l index score (27). Using this model, the EQ-5D-3l health index spans from 1.00 for the best possible health state to −0.38 for the worst possible health state.

### 2.7 Features and outcomes

The analyzed dataset includes adherence to three intervention categories (diet, PA, and vitamin D Supplementation) recorded at three time points during the first year of the program.

In addition to the temporal adherence measures, the dataset includes at BL a comprehensive set of clinical characteristics, which serves as key variables for understanding patient profiles. These characteristics are shown in Supplementary Table S1. Compliance with diet, exercise, and vitamin D supplementation was assessed based on data collected up to M12. We then predicted the most common compliance behavior across the three follow-up points in the next 9 months. For HRQoL prediction, we used scores from BL to M12, considering baseline characteristics, and then predicted the average HRQoL score for the following 9 months.

## 3 Statistical analysis

All the computational and statistical analysis were performed using Python 3.10.6 [https://www.python.org/downloads/release/python-3106]. A complete list of the Python packages and libraries employed, along with their respective versions, is provided in Supplementary Table S1.

### 3.1 Data preprocessing

The initial analysis focused on the entire trajectories of HRQoL scores among patients, revealing a significant amount of missing data across the 12 quarterly measurements. Initially, 492 subjects were included in the study. Among them, 176 were excluded because they had more than 4 missing values in their HRQoL trajectory, a criterion adopted to ensure the reliability of the imputations and to reduce potential bias. Most subjects had no missing data, followed by those with 1, 2, or 3 missing values.

A more restrictive threshold than the one adopted (e.g., 3 missing values) would have led to the exclusion of a substantial number of subjects, significantly reducing the amount of data available for model training. To impute missing values, we used the 93 fully observed cases to train and test various imputation models. The model that achieved the best performance on the test set (shown in Table 1) was then applied during the inference phase to estimate the missing values in the remaining 223 subjects. Following imputation, 316 complete cases were obtained. While it is well known in the literature that imputation may introduce some degree of bias, in real-world scenarios where ideal, (gold standard) data are not available, it remains a valuable approach to enable the development and validation of predictive ML and Deep Learning models (28). Specifically, we employed the Iterative Imputer with an Extra Trees Regressor estimator (29) due to its ability to handle non-linear relationships within the data. The method iteratively predicts missing values by modeling each incomplete feature as a function of the other features. This process repeats until the imputations stabilize, ensuring consistent and reliable estimates. To assess the performance of this approach, we compared it with several other imputation methods, including Iterative Imputer with a Bayesian Ridge estimator (30), K-Nearest Neighbors (KNN) Imputer (31), and Simple Imputer (mean strategy) (32). Each method was implemented and evaluated using the Root Mean Square Error (RMSE) and the Coefficient of Determination (*R*^2^), which are explained more in detail in the paragraph 3.3. These metrics calculated between the imputed values and the observed values in complete cases.

**Table 1:** Summary of the imputation methods.

Imputation Methods	RMSE	*R* ^2^
Iterative Imputer (Bayesian Ridge)	0.55	0.66
K-Nearest Neighbors (KNN) Imputer	0.61	0.55
Simple Imputer (Mean Strategy)	0.58	0.56
**Iterative Imputer (Extra Trees Regressor)**	**0**.**41**	**0**.**75**

RMSE, Root Mean Squared Error; *R*^2^, coefficient of determination.

The best algorithm is highlighted in bold. RMSE measures prediction accuracy, with smaller values indicating better performance, the coefficient of determination (*R*^2^) indicates the proportion of variance explained, with larger values indicating better performance.

### 3.2 Clustering—QoL group identification

Following the imputation process, hierarchical clustering was performed on the imputed HRQoL trajectories to explore potential differences over the follow-up period (33). Specifically, a divisive hierarchical clustering approach was used. The clustering analysis identified a majority group, which represented the most common pattern in the HRQoL trajectories. The Mann–Whitney *U*-test was used to test the null hypothesis of non-different means between groups with a 95% significance level. Results revealed significant differences in the slopes of the HRQoL trajectories between this majority cluster and the other identified groups. These findings highlighted the majority cluster as the primary focus for subsequent analysis, enabling a more targeted exploration of the dominant HRQoL trends. A *post-hoc* analysis was assessed to quantify the proportion of patients with imputed data within each cluster to evaluate whether the clustering solution might have been influenced by data completeness.

### 3.3 Forecasting models

To answer RQ1, we approached the adherence prediction as a multi-label time series classification task, with patient compliance for each advice category (diet, PA, and supplemental vitamin D) recorded at each trimester on a discrete scale of 1–3. Specifically, three separate Random Forest classifier (RF) models were developed, each one for a single specific outcome: future adherence to one of the three recommendations. These models utilized a comprehensive input dataset, which included temporal adherence patterns for diet, PA, and vitamin D supplementation recorded during the first year of the program (M3, M6, M9, and M12), combined with BL characteristics (age, cancer stage, molecular subtypes, radio-, chemo-, neoadjuvant therapy status, comorbidities). The target label for each classifier represented the most frequently recorded adherence value in its respective category during the subsequent nine-month period and was treated as a classification problem. Although the input data remained constant across models, each classifier was tasked with predicting a different target variable, specifically the future adherence to one of the three recommendations. To answer RQ2, we employed an XGBoost, a gradient boosting regressor, chosen for its effectiveness with continuous outcomes (34). This algorithm is part of supervised regression ensemble models adopting a Decision Tree (DT) sequence: the algorithm works by adding a new DT to the former ones to minimize the regression error, which was the residue of that series. XGBoost incorporated BL values and QoL time series data from the program's first year to forecast the average QoL score by the one-year treatment. A linear regression (LR) model was fitted as a reference algorithm to compare the performances of the former one. We performed a 70/30% train/test split on our dataset to prepare it for model training and evaluation. This split allowed us to use 70% of the data for training the models and 30% for testing their performance on unseen data. All samples were uniquely assigned to either the training or the test set. The split was carried out randomly to avoid selection bias, ensuring that the distribution of the target variable was preserved across training and test sets. We adopted a k-fold cross validation on the training data to assess the best tuning of algorithms, namely that set of hyperparameters, chose from a wide possibility of combinations (often set in a grid), which are associated with the highest performances. For each combination of hyperparameters, data are split into random k equal folds of observations: each part is further divided into *k* − 1 parts dedicated to fit the algorithm while the kth part is used to measure its performances. The best combination of hyperparameters is the one to be finally chose for the final algorithm. We used *k* = 5 as default value from the sci-kit learn method in Python. A fixed random seed was applied to ensure the reproducibility of the experiments.

After training and optimizing our models, we evaluated their performance on the test set that was held out during the initial split. Main metrics were used for performance assessment. Precision, Recall, F1-score and Accuracy were calculated for classification problems. Precision measures the accuracy of correctly labeled predictions, indicating how many of the predicted labels were correct. Recall represents the ratio of correctly detected labels out of all the true labels. The F1-score provides a balance between precision and recall, calculated as the harmonic mean of these two metrics. It is defined as the ratio of true positives to the sum of true positives and the average number of misclassified labels. These measures are typically used for classification problems. RMSE, mean absolute error (MAE), mean squared error (MSE), *R*^2^ and The Bland-Altman plots were performed to analyze the quality from regression models (35). For assessing prediction quality on continuous data, MAE is a common metric used in regression tasks, providing the mean of absolute differences between predictions and actual values. However, it is an absolute measure, meaning it does not allow direct comparison between models with different units of measurement or different value ranges (36, 37). To address this issue, coefficient of determination (*R*^2^), ranging from zero to one, was also considered. Moreover, we adopted the RMSE which gives a measure of the standard deviation of errors and penalizes larger errors more heavily than MAE, which treats errors linearly, making it less sensitive to outliers (36–38). The choice of evaluation metric depends on the application context and the data: to penalize large errors, RMSE is preferable when large errors need to be penalized, while MAE is better for a more robust approach to outliers' detection.

### 3.4 Feature importance

Computing the Feature Importance (FI) is a convenient technique to rank the included features in terms of association with the outcome of interest (39). Both the adopted algorithms allowed such a function, so importance scores were extracted from the RF classifiers and the XGBoost regressor to identify the key variables contributing to the predictions. For the RFs, these scores were calculated using the mean decrease in Gini impurity, allowing us to rank the features based on their influence on the classification outcomes. In the XGBoost regression model, used for HRQoL predictions, FI was evaluated based on the gain, which measures the improvement in accuracy brought by a feature to the branches it appears in. Plots assessing the importance scores of each feature were shown and the most important variable were listed and commented. Additionally, *R*^2^ provides a measure of the model's goodness of fit, though it may not always be sufficient for a complete evaluation of performance (40, 41). All the computational and statistical analyses were performed using Python 3.10.6 [https://www.python.org/downloads/release/python-3106].

## 4 Results

### 4.1 Missing imputation

A total of 316 BC patients were included in the analysis. Table 1 summarizes the performance of four different imputation techniques applied to the longitudinal trajectories of HRQoL scores, aimed at handling missing data (Iterative Imputer with Extra Trees Regressor, Iterative Imputer with Bayesian Ridge Regressor, KNN Imputer, Simple Imputer). The method based on the Extra Trees Regressor showed the best performance (RMSE of 0.41 and the *R*^2^ of 0.75), demonstrating higher accuracy compared to the alternative approaches.

### 4.2 Clustering

**Figure 1:**
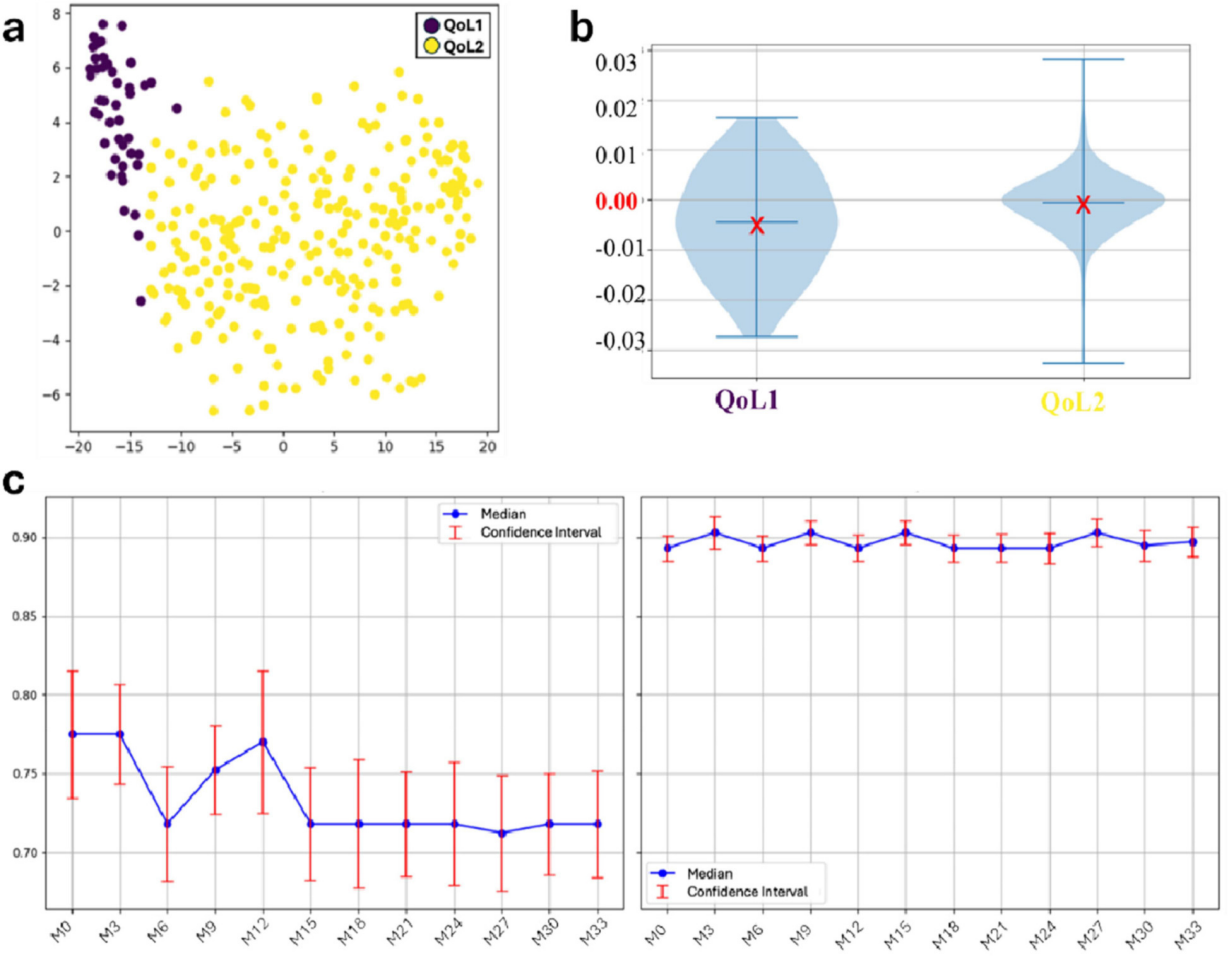
Results from clustering algorithm and trend analysis among the two HRQoL groups throughout the 9 months period: **(a)** scatter plot; **(b)** HRQoL slope distribution by groups. The red X marks the mean slope within each group: negative mean values indicate a decreasing trend over time, whereas values close to zero suggest overall stability. **(c)** QoL trends among the groups. The red bounded bars represent the 95% confidence interval for each single mean point. After imputing missing values, all the HrQoL measures were used for clusering. QoL, Quality of Life; HRQoL, Health Related Quality of Life.

Following imputation, the hierarchical clustering algorithm automatically detected two main QoL-oriented groups (QoL1 and QoL2) (Figure 1a). QoL1 (*n* = 45) showed more variability compared to QoL2 (*n* = 271) (Figure 1b); regarding trends, the QoL2 group showed greater stability and higher scores (Figure 1c) and it was adopted for our further analysis. The Mann–Whitney *U*-test confirmed statistically significant differences between the two clusters (*p* *<* 0*.*001). The *post-hoc* analysis revealed that 80% of patients in QoL1% and 69% in QoL2 had imputed values. The relatively modest difference between the two groups supported the validity of the clustering approach, demonstrating that the algorithm identified patient groups based on HRQoL patterns rather than data completeness. Table 2 provides a detailed comparison between the two QoL clusters in terms of clinical profiles, highlighting distinctive patterns among patients with different HRQoL trajectories.

**Table 2:** Distribution of BL characteristics between quality of life (QoL) clusters.

Feature	QoL1 (*n* = 45)	QoL2 (*n* = 271)
Age (years)		
Median (Q1–Q3)	50 (46–57)	55 (49–62)
Smoking status		
No smoking	20 (44.6%)	115 (42.2%)
Smoker	10 (22.9%)	72 (26.7%)
Former Smoker	15 (32.5%)	84 (31.1%)
Number of comorbidities		
0	26 (59%)	187 (68.9%)
1	12 (25.8%)	36 (13.3%)
2	5 (10.0%)	36 (13.3%)
3	2 (4.1%)	6 (2.2%)
4+	1 (1.1%)	6 (2.2%)
Cancer Stage		
I	13 (28.4%)	72 (26.7%)
IIA	20 (45.0%)	102 (37.8%)
IIB	5 (11.8%)	54 (20%)
IIIA	4 (10.3%)	42 (15.6%)
IIIC	2 (4.4%)	0 (0.0%)
Lymph node status		
N0	21 (46.9%)	114 (42.2%)
N1	17 (38.4%)	114 (42.2%)
N2	5 (10.3%)	42 (15.6%)
N3	2 (4.4%)	0 (0.0%)
Tumor size		
T1	28 (61.6%)	157 (57.8%)
T2	17 (38.0%)	108 (40.0%)
T3	1 (0.4%)	6 (2.2%)
Molecular subtypes		
Luminal A	15 (34.3%)	78 (28.9%)
Luminal B	21 (46.1%)	138 (51.1%)
HER2+	2 (4.4%)	12 (4.4%)
TN	7 (15.1%)	42 (15.6%)
Radiotherapy status		
Never	16 (34.6%)	123 (45.5%)
Ongoing	4 (9.8%)	18 (6.8%)
Finished	25 (55.6%)	129 (47.7%)
Chemotherapy status		
Never	16 (36.2%)	102 (37.8%)
Completed (<2 months)	7 (15.9%)	42 (15.6%)
Completed (≥2 months)	14 (30.6%)	78 (28.9%)
Ongoing	8 (17.3%)	48 (17.8%)
Neoadjuvant therapy status		
Never	40 (88.9%)	253 (93.3%)
Finished	5 (11.1%)	18 (6.7%)
Type of surgery		
Quadrantectomy	35 (77.5%)	203 (75.0%)
Mastectomy	10 (22.5%)	68 (25.0%)
Time from surgical intervention (months)		
Median (Q1–Q3)	7 (5–10)	8 (5–10)

Numbers may not sum up to the *n*'s from QoL clusters due to the presence of missing values, subsequently treated. Data are presented as median (interquartile range, 25th–75th percentile) for numerical variables, and as absolute numbers for categorical variables. The number of comorbidities is a cumulative measure of pre-existing health conditions that reflects the overall burden of comorbidities. It includes type 1 and type 2 diabetes, hypertension, hypertriglyceridemia, hypercholesterolemia, and hyperglycemia. Additionally, it accounts for cancer stage, which represents the extent of BC at baseline. Chemotherapy status (chemo status) is classified as completed (<2 months or ≥2 months), ongoing, or never initiated. Molecular subtypes, derived from receptor status (ER, PgR, HER2), are used to classify the intrinsic molecular subtypes of BC. Finally, neoadjuvant chemotherapy status is categorized as completed or never initiated, indicating whether neoadjuvant chemotherapy was finished prior to surgery.

QoL, Quality of Life; ER, estrogen receptors; PgR, progesterone receptors; HER2, Human Epidermal Growth Factor Receptor 2; TN, Triple-Negative.

### 4.3 Forecasting the compliance to the treatment

The optimal hyperparameters identified for the three RFs and the XGBoost model used for the prediction task are summarized in Supplementary Tables S2a,b. The evaluation metrics for the three RFs trained to predict future AD_DIET, AD_PA, and AD_VITD are summarized in Table 3. The highest accuracy was achieved by the model predicting AD_DIET (0.81), followed by the model for AD_VITD (0.79) and AD_PA (0.71).

**Table 3:** Performance of RFs in predicting future adherence to the three recommendations: each row corresponds to a single RF trained to predict adherence to one of the three recommendations.

Adherence	Precision	Recall	F1-Score	Accuracy
Diet	0.79	0.80	0.80	0.81
Physical Activity	0.81	0.77	0.79	0.71
Vitamin D	0.77	0.78	0.77	0.79

RF, Random Forest.

The reported metrics are macro-averages, ensuring robustness to class imbalance.

**Figure 2:**
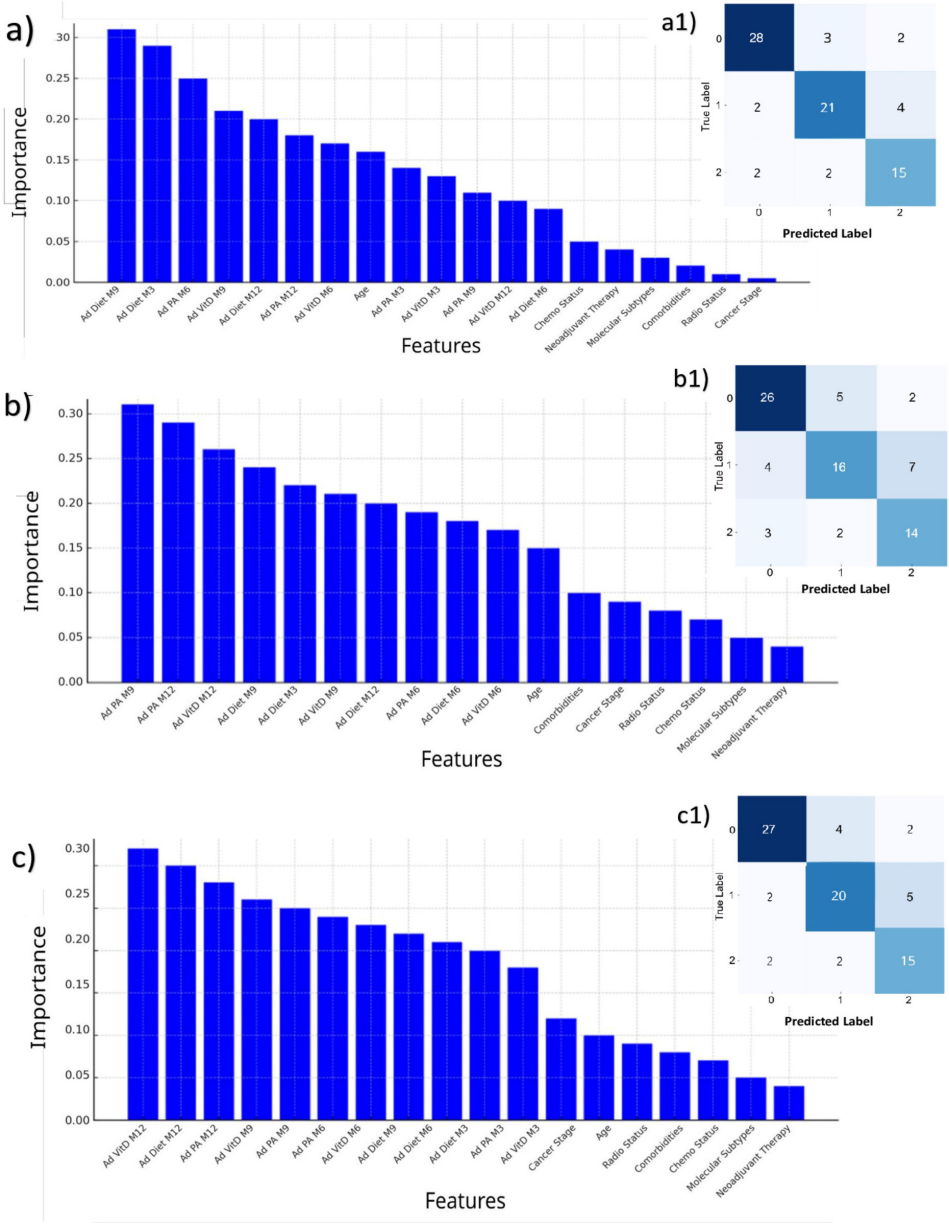
Feature importances (left) and prediction results (right) of the three RFs, for predicting the most frequent value over the next 9 months in adherence to dietary advice **(a,a1)**; physical activity **(b,b1)**; supplemental vitamin D **(c,c1)**. In each confusion matrix, the classes labeled as 0, 1, and 2 correspond to adherence levels 1, 2, and 3, respectively. Each class represents the adherence level most frequently observed over the subsequent 9 months. RF, Random Forest.

**Figure 3:**
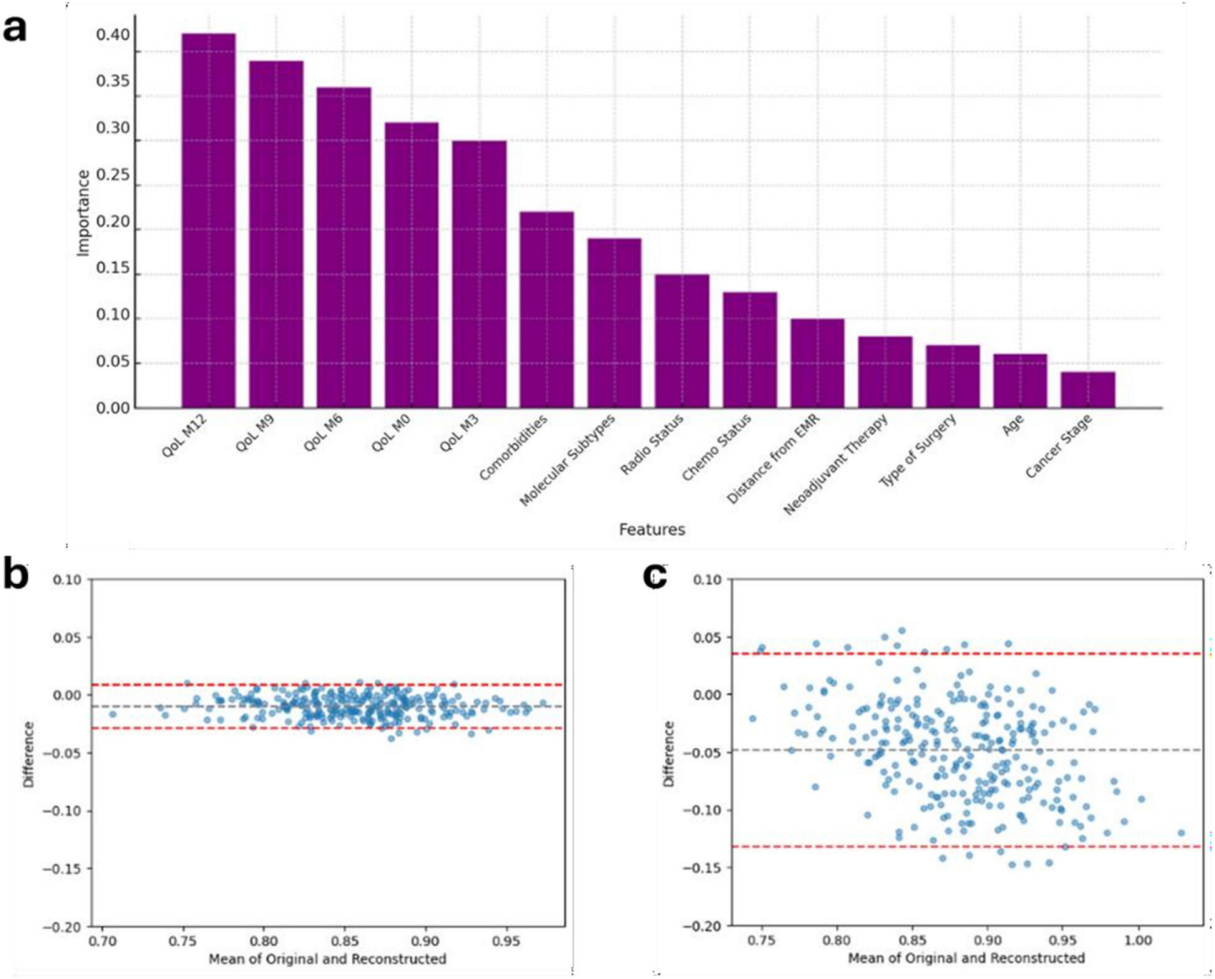
Results from the XGBoost model. **(a)** Feature importance plot illustrating the most influential predictors for estimating mean QoL scores over the subsequent 9 months of clinical follow-up. **(b)** Bland–Altman plot for the XGBoost model, and **(c)** Bland–Altman plot for LR model, provide a comparative assessment of agreement between predicted and observed HRQoL scores. Each dot represents a subject; the black dashed line indicates the mean difference (bias), and the red dashed lines the 95% limits of agreement, providing establish if accordance between predicted and observed values (subjects) statistically exists. LR, linear regression; QoL, Quality of Life; HRQoL, Health releated QoL.

On the right side of Figure 2, the confusion matrices are shown. In all three models, the BL characteristics were found to be less related to the outcomes. A comparison between the XGBoost model and the multivariable LR on predicting the mean HRQoL score over the following 9 months is presented in Table 4. The XGBoost model demonstrated superior performance across all metrics, with an *R*^2^ of 0.62 compared to 0.42 for the LR model, underscoring the enhanced predictive capability of non-linear methods in modeling complex health outcomes. Notably, the Bland-Altman analysis (Figures 3b,c) revealed a strong difference in predictions between XGBoost and LR. Specifically, plot Figure 3b shows a good agreement without systematic drift, with minimal differences randomly distributed around the mean difference line; plot Figure 3c exhibits a negative drift, indicating that the model tends to underestimate predictions as mean of the measurements increases.

**Table 4:** Performances measures from XGboost and LR.

Model	RMSE	MAE	MSE	*R* ^2^
XGBoost	0.04	**0** **.** **03**	**0** **.** **002**	**0** **.** **62**
LR	0.04	0.06	0.005	0.42

LR, linear regression; MAE, Mean Absolute Error; MSE, Mean Squared Error; RMSE, Root Mean Squared Error.

The best model is highlighted in bold, indicating the one with better prediction accuracy, as smaller values mean having a better performance. The coefficient of determination (*R*^2^) indicates the proportion of variance explained, with larger values indicating better performance.

### 4.4 Important features

The important score reflects the impact of each feature on enhancing the model accuracy in predicting outcomes (Figure 2, left hand). Diet adherence was mainly influenced by its M9 and M3 components, respectively, and by M6 adherence to PA (Figures 2a,a1). Adherence to PA was principally predicted by its closer components, i.e., M9 and M12 adherence to PA, and by M12 adherence to vitamin D (Figures 2b,b1). All the M12 adherence measures were found as the features, which mostly were related to the adherence to vitamin D (Figures 2c,c1). Most important features contributing to the HRQoL average prediction were the 5 measures throughout the year of HRQoL; the number of comorbidities was found as the most ranked first BL characteristic out of HRQoL lags (Figure 3a).

## 5 Discussion

Our goal was to apply ML algorithms, to model and predict health-related behaviors in women diagnosed with BC who were enrolled in the DEDiCa study, a lifestyle modification clinical trial that followed participants for three years during treatment involving diet, PA, and vitamin D supplementation. Clinical studies have provided substantial evidence supporting the effectiveness of targeted lifestyle interventions. A systematic review published in 2022 assessed the impact of physical and nutritional interventions in BC patients, concluding that an integrated program combining physical activity and diet can reduce the risk of relapse and enhance quality of life (42).

Regular physical exercise contributes to reducing the risk of recurrence, improving survival, energy, sleep quality, mental health, and reducing anxiety, depression, and fatigue (43–46). Similarly, nutritional interventions play a key role before, during, and after cancer treatment. A balanced and adequate diet, rich in essential nutrients and antioxidants, such as the Mediterranean diet, can contribute to improving immune function and reducing the risk of comorbidities, such as diabetes or cardiovascular diseases, frequently observed in patients with BC (47). Promoting a Mediterranean diet can improve metabolic health and reduce chronic inflammation in these patients, as well as improving the response to oncological treatments, and is also associated with a better QoL and a lower incidence of BC recurrence (48, 49). Porciello et al. in two studies showed that adherence to the Mediterranean diet and a high-quality diet (according to the Healthy Eating Index (HEI-2015 index) are associated with significant improvements in QoL particularly in terms of physical functioning, pain, and overall wellbeing (20, 21). The inclusion of PA and therapeutic phase in the analysis enhances the understanding of the factors influencing QoL in this context. These results showed the importance of integrated, multidisciplinary interventions combining nutritional strategies and PA within follow-up programs for BC survivors (20, 21). Nevertheless, while these studies provide robust evidence, traditional statistical approaches may fail to capture the complexity and individual variability of QoL trajectories over time.

In this context, our study introduces an innovative contribution through the application of ML models, offering a dynamic, predictive, and interpretive dimension to the analysis of lifestyle interventions in oncology. From these multiple investigations, conducted on a cohort of 316 patients the FI framework detected the presence of underlying associations between diet quality, PA, and HRQoL, while providing new insights into the temporal patterns of these relationships.

Through the integration of ML methodologies, this research complements existing evidence and advances the personalization of supportive care strategies in BC survivorship. In particular, the RF and XGBoost models demonstrated superior performance compared to traditional approaches, showing greater accuracy in predicting average HRQoL levels. Moreover, these models effectively identified the behavioral factors that most strongly influence HRQoL trajectories during follow-up. Our analyses revealed that repeated measurements over time of diet, PA, and vitamin D supplementation are significantly more relevant predictors than baseline variables, thus highlighting the importance of continuous and personalized monitoring throughout the care pathway. A major challenge in longitudinal clinical studies is managing missing data due to patient dropouts (50): in this regard, we found that only 93 patients reported complete data, at least in HRQoL measure. By employing four ML algorithms for missing data imputation, we found that the Iterative Imputer outperformed the other methods, including the Simple Imputer, despite this is the most commonly used technique for imputing missing data in diet-related studies. Furthermore, this analysis identified the key factors influencing patient adherence to the lifestyle program and future HRQoL outcomes, offering valuable insights into the complex interactions of variables across the different predictive models. This approach allowed for a comprehensive analysis of both adherence to lifestyle program and HRQoL trajectories during the initial phase of the intervention.

We selected a more stable subset of patients in terms of HRQoL behavior throughout the follow-up period, we focused on the patient's compliance to the treatment. We aimed to predict the most frequent value over the next 9 months after one year of intervention in adherence to diet, PA, and vitamin D supplementation: PA was harder to depict based on baseline characteristics and its four quarter lags (accuracy = 0.71) than vitamin D (accuracy = 0.79) or diet (accuracy = 0.81). Among our analyses, we found out that baseline characteristics played a secondary role on predictions, with the age almost the first ranked feature among them. In particular, the most frequent value of vitamin D was quite mostly associated with the M12 measures compared to the advice regarding diet and PA. Secondly, the HRQoL score was analyzed in terms of mean value over the subsequent 9 months of follow-up after one year of intervention by adopting the XGBoost regressor and a multivariable LR model.

In this context, the boosting method revealed a much higher performance compared to the linear model, with a plus 20 points-percent in *R*^2^ statistics. This finding suggested a likely higher power of such method to predict the outcome on well-known methods. Indeed, it was confirmed from the Bland-Altman plot that the latter model showed an underestimation of HRQoL mean value. This interesting result may confirm the non-linearity relation between the outcome and the set of features.

## 6 Limitations

This predictive analysis has several limitations. First, since the dataset used for model training and evaluation had a relatively small sample size, especially after excluding patients with significant missing data, this gap may limit the generalizability of the findings. Second, despite advance imputation techniques being employed to handle missing data, imputation methods may introduce biases. It is more evident when data are not missing completely at random.

Alternative methods like Multiple Imputation by Chained Equations could have been considered. However, this approach was not implemented because it assumes parametric relationships between variables, which may not adequately capture the non-linear interactions present in this dataset. Third, the algorithms used for ML modelling (i.e., RF and XGBoost), although effective, may not fully capture complex temporal dependencies or interactions between features over time. For multi-label classification, algorithms like gradient boosting machines might have delivered better performance with a larger dataset. A similar challenge was observed in the cluster analysis. With a larger dataset, we could have improved the temporal analysis by utilizing techniques like K-Shape or density-based clustering (DBSCAN) to better handle groups with non-uniform distributions. Additionally, another key limitation of this study is the lack of a thorough explainability analysis. The use of more advanced explainability methods, such as SHapley Additive exPlanations (known as the SHAP method), could have enhanced the interpretability of the models' predictions.

Lastly, the analysis relied on a 3-level only adherence scale based on operators' perception and on a single cohort from a specific geographic and clinical context limiting the applicability of the results to other populations. Future studies should aim to validate these findings on larger, more diverse datasets and to better evaluate the models' performances in real world scenarios.

## 7 Conclusions

The findings of our study underscore the significant potential of ML algorithms in advancing personalized healthcare for BC survivors. By predicting adherence to lifestyle recommendations and forecasting QoL outcomes, these tools provide critical insights into patients' health behaviors and trajectories. The study itself revealed that adherence to dietary advice and vitamin D supplementation could be predicted with higher accuracy compared to physical activity, emphasizing the complexity of the latter. Additionally, the superior performance of XGBoost in QoL prediction highlights the value of employing advanced regression techniques for non-linear relationships in health data. Key insights include the pivotal role of adherence measures at specific time points (e.g., M12) in influencing predictions, as well as the limited but notable influence of baseline characteristics. These findings suggest that interventions focusing on consistent and measurable adherence behaviors are essential for optimizing long-term outcomes.

Future applications of this work could expand beyond BC to other chronic conditions where lifestyle modifications play a crucial role and integrating ML-driven insights into clinical practice to support healthcare providers in developing targeted and effective intervention strategies, ultimately improving patient QoL. Clinical prediction has gained greater importance in modern healthcare, involving the use of medical data to forecast future health outcomes. This process is applied in various fields of disease, from prevention to diagnosis and treatment resulting in better patient outcomes and improved efficiency of healthcare systems. ML algorithms can rapidly analyze large, complex medical data with high precision, detecting patterns and correlations that might be beyond the scope of human analysis. When feed with temporal data, they can be designed to continuously learn from new data, improving their predictive accuracy over time (51). To the best of our knowledge, few studies have aimed to predict future patient compliance with lifestyle programs using ML approaches. Mousavi et al. (52) implemented, a hybrid model combining artificial neural network and genetic algorithm to predict adherence to diet among patients referred to a private clinic in Iran, leading to high accuracy in predicting diet adherence (93.5%) and proper performance. Regarding feature importance, a genetic algorithm selected some patients-related factors that could affect diet adherence, including weight, weight satisfaction, and body mass index, lunch, dinner and sleep time. The implementation of this model in the clinical practice could be useful to identify patients with low chance of diet adherence, supporting dietitians to employ the proper nutritional strategy (52). Similarly, in the study of Kim et al. (53), seven ML algorithms were implemented to predict QoL in middle-aged South Korean adults. The RF method showed the highest performance in predicting QoL deterioration and the highest performance. Regarding feature importance, the authors showed that sleep quality and stress were identified as the most important predictors of QoL by the model.

## Data Availability

The original data presented in this study will be available upon request to the last author (LA, l.augustin@istitutotumori.na.it) and for research purposes only (https://zenodo.org/records/15601115).
