# Correction: Adopting machine learning to predict breast cancer patients adherence with lifestyle recommendations and quality of life outcomes

**DOI:** 10.3389/fdgth.2026.1804768

**Published:** 2026-03-11

**Authors:** Anna Crispo, Maria Elisabetta Pagnano, Agnese Bonfigli, Leandro Pecchia, Assunta Luongo, Giuseppe Porciello, Sergio Coluccia, Melania Prete, Luca Bacco, Sara Vitale, Elvira Palumbo, Paolo Giaccone, Rosa Pica, Maria Grimaldi, Marco Cascella, Ernesta Cavalcanti, Anita Minopoli, Michelino De Laurentiis, Massimo Libra, Jerry Polesel, Samuele Massarut, Egidio Celentano, Livia S. A. Augustin

**Affiliations:** 1 Epidemiology and Biostatistics Unit, Istituto Nazionale Tumori - IRCCS, “Fondazione G. Pascale”, Naples, Italy; 2 Research Unit of Intelligent Health-Technologies, Department of Engineering, Università Campus Bio-Medico di Roma, Rome, Italy; 3 Fondazione Policlinico Universitario Campus Bio-Medico, Rome, Italy; 4 Branch of Medical Statistics, Biometry and Epidemiology “G. A. Maccacaro”, Department of Clinical Sciences and Community Health, Università degli Studi di Milano, Milan, Italy; 5 Research Unit of Computer System and Bioinformatics, Department of Engineering, Università Campus Bio-Medico di Roma, Rome, Italy; 6 Anesthesia and Pain Medicine, Department of Medicine, Surgery and Dentistry “Scuola Medica Salernitana”, University of Salerno, Baronissi, Italy; 7 Division of Laboratory Medicine, Istituto Nazionale Tumori - IRCCS, “Fondazione G. Pascale”, Naples, Italy; 8 Division of Breast Medical Oncology, Department of Breast and Thoracic Oncology Director, Istituto Nazionale Tumori - IRCCS, “Fondazione G. Pascale”, Naples, Italy; 9 Department of Biomedical and Biotechnological Sciences, University of Catania, Catania, Italy; 10 Cancer Epidemiology Unit, Centro di Riferimento Oncologico di Aviano (CRO), IRCCS, Aviano, Italy; 11 Breast Surgical Oncology Unit, Centro di Riferimento Oncologico di Aviano (CRO), IRCCS, Aviano, Italy

**Keywords:** breast cancer, machine learning, missing data, diet, health-related quality of life

The Data Availability statement was erroneously given as “The original data presented in this study will be available upon request. Requests to access the datasets should be directed to the last author (LA), l.augustin@istitutotumori.na.it.” The correct Data Availability statement is “The original data presented in this study will be available upon request to the last author (LA, l.augustin@istitutotumori.na.it) and for research purposes only (https://zenodo.org/records/15601115).”

The original version of this article has been updated.

