# Supplementary material for: Adopting machine learning to predict breast cancer patients adherence with lifestyle recommendations and quality of life outcomes
Source: Front Digit Health. 2025 Nov 6;7:1645233. doi: 10.3389/fdgth.2025.1645233 (PMC12631627; doi:10.3389/fdgth.2025.1645233)
Supplement: Supplementary file 1 [file Datasheet1.docx]

Supplemental material

| Hyperparameter | Property | RF Diet |
| --- | --- | --- |
| **Package** | **Library (import)** | **Version** |
| NumPy [1] | `numpy` | 1.23.5 |
| Pandas [2] | `pandas` | 1.5.3 |
| Matplotlib [3] | `matplotlib.pyplot` | 3.6.3 |
| Seaborn [4] | `seaborn` | 0.12.2 |
| SciPy [5] | `scipy.cluster`, `scipy.spatial` | 1.10.1 |
| Scikit-learn [6] | `sklearn.*` (impute, ensemble, linear_model, tree, metrics, cluster, model_selection) | 1.2.2 |
| FastDTW [7] | `fastdtw` | 0.3.4 |
| Tslearn [8] | `tslearn.metrics` | 0.5.3.2 |
| XGBoost [9] | `xgboost` | 1.7.6 |
| Random Forest¹ [10] | `sklearn.ensemble.RandomForestClassifier` | included in scikit-learn 1.2.2 |

**Table s1**: List of Python packages and libraries used in this study, with corresponding imports and version numbers

| Hyperparameter |  | RF Diet | RF Vit D | RF PA |
| --- | --- | --- | --- | --- |
| n estimators | Number of trees | 200 | 150 | 250 |
| max depth | Maximum depth of a tree | 10 | 15 | 12 |
| min samples split | Minimum number of samples required to split an internal node | 5 | 4 | 6 |
| min samples leaf | Minimum number of samples required to be at a leaf node | 2 | 3 | 1 |
| max features | Number of features to consider when looking for the best split | sqrt | log2 | auto |
| bootstrap | Random subsampling used when building trees | True | false | true |
| class weight | Imbalance treatment with class weights | balanced subsample | balanced | none |

**Table s2a:** Optimal hyperparameters identified for the RF models.

**Abbreviation:** RF, Random Forest

| **Hyperparameter** | **XGBoost** |
| --- | --- |
| learning_rate | 0.1 |
| n_estimators | 300 |
| max_depth | 5 |
| subsample | 0.8 |
| colsample_by tree | 0.8 |
| gamma | 0.1 |
| alpha | 0.1 |
| lambda | 1.5 |

**Table s2b:** Optimal hyperparameters identified for the XGBoost model.
