# Supplementary material for: Adopting machine learning to predict breast cancer patients adherence with lifestyle recommendations and quality of life outcomes
Source: Front Digit Health. 2025 Nov 6;7:1645233. doi: 10.3389/fdgth.2025.1645233 (PMC12631627; doi:10.3389/fdgth.2025.1645233)
Supplement: Supplementary file 2 [file Supplementaryfile1.docx]

1. Harris CR, Millman KJ, van der Walt SJ, Gommers R, Virtanen P, Cournapeau D, Wieser E, Taylor J, Berg S, Smith NJ, Kern R, Picus M, Hoyer S, van Kerkwijk MH, Brett M, Haldane A, Del Río JF, Wiebe M, Peterson P, Gérard-Marchant P, Sheppard K, Reddy T, Weckesser W, Abbasi H, Gohlke C, Oliphant TE. Array programming with NumPy. Nature. 2020 Sep;585(7825):357-362. doi: 10.1038/s41586-020-2649-2. Epub 2020 Sep 16. PMID: 32939066; PMCID: PMC7759461.
2. McKinney, W. (2010) Data Structures for Statistical Computing in Python. Proceedings of the 9th Python in Science Conference, Austin, 28 June-3 July 2010, 56-61.
   <https://doi.org/10.25080/Majora-92bf1922-00a>
3. Hunter, J.D. (2007) Matplotlib: A 2D Graphics Environment. Computing in Science & Engineering, 9, 90-95.
   <http://dx.doi.org/10.1109/MCSE.2007.55>
4. Waskom, M. L., (2021). seaborn: statistical data visualization. Journal of Open Source Software, 6(60), 3021, <https://doi.org/10.21105/joss.03021>
5. Virtanen P, Gommers R, Oliphant TE, Haberland M, Reddy T, Cournapeau D, Burovski E, Peterson P, Weckesser W, Bright J, van der Walt SJ, Brett M, Wilson J, Millman KJ, Mayorov N, Nelson ARJ, Jones E, Kern R, Larson E, Carey CJ, Polat İ, Feng Y, Moore EW, VanderPlas J, Laxalde D, Perktold J, Cimrman R, Henriksen I, Quintero EA, Harris CR, Archibald AM, Ribeiro AH, Pedregosa F, van Mulbregt P; SciPy 1.0 Contributors. SciPy 1.0: fundamental algorithms for scientific computing in Python. Nat Methods. 2020 Mar;17(3):261-272. doi: 10.1038/s41592-019-0686-2. Epub 2020 Feb 3. Erratum in: Nat Methods. 2020 Mar;17(3):352. doi: 10.1038/s41592-020-0772-5. PMID: 32015543; PMCID: PMC7056644.
6. Pedregosa, F., Varoquaux, G., Gramfort, A., et al. (2011) Scikit-Learn: Machine Learning in Python. Journal of Machine Learning Research, 12, 2825-2830.
7. Salvador S, Chan P: FastDTW: Toward accurate dynamic time warping in linear time and 725 space. Proceedings of the 10th ACM SIGKDD Workshop on Mining Temporal and Sequential Data 726 2004, 70–80. 727
8. Romain Tavenard, Johann Faouzi, Gilles Vandewiele, Felix Divo, Guillaume Androz, Chester Holtz, Marie Payne, Roman Yurchak, Marc Rußwurm, Kushal Kolar, and Eli Woods. 2020. Tslearn, a machine learning toolkit for time series data. J. Mach. Learn. Res. 21, 1, Article 118 (January 2020), 6.
9. Chen T, Guestrin C: XGBoost: A scalable tree boosting system. Proceedings of the 22nd 730 ACM SIGKDD International Conference on Knowledge Discovery and Data Mining 2016, 785– 731 794. 732
10. Breiman L: Random forests. Machine Learning 2001, 45(1):5–32.
